# Critical Evaluation of Molecular Monitoring in Malaria Drug Efficacy Trials and Pitfalls of Length-Polymorphic Markers

**DOI:** 10.1128/AAC.01500-16

**Published:** 2016-12-27

**Authors:** Camilla Messerli, Natalie E. Hofmann, Hans-Peter Beck, Ingrid Felger

**Affiliations:** a Swiss Tropical and Public Health Institute, Basel, Switzerland; b University of Basel, Basel, Switzerland

**Keywords:** genotyping, PCR, Plasmodium falciparum, amplification bias, drug trial, *glurp*, *msp1*, *msp2*, template competition

## Abstract

Estimation of drug efficacy in antimalarial drug trials requires parasite genotyping to distinguish new infections from treatment failures. When using length-polymorphic molecular markers, preferential amplification of short fragments can compromise detection of coinfections, potentially leading to misclassification of treatment outcome. We quantified minority clone detectability and competition among *msp1*, *msp2*, and *glurp* amplicons using mixtures of Plasmodium falciparum strains and investigated the impact of template competition on genotyping outcomes in 44 paired field samples. Substantial amplification bias was detected for all three markers, with shorter fragments outperforming larger fragments. The strongest template competition was observed for the marker *glurp*. Detection of *glurp* fragments in multiclonal infections was severely compromised. Eight of 44 sample pairs were identified as new infections by all three markers. Ten pairs were defined as new infections based on one marker alone, seven of which were defined by the questionable marker *glurp*. The impact of size-dependent template competition on genotyping outcomes therefore calls for necessary amendments to the current WHO recommendations for PCR correction of malaria drug trial endpoints. Accuracy of genotyping outcomes could be improved by separate amplification reactions per allelic family and basing results on markers *msp1* and *msp2* first, with *glurp* only used to resolve discordant results.

## INTRODUCTION

In areas of high malaria endemicity, individuals are often multiply infected with Plasmodium falciparum. The number of concurrent clones per infected individual is denoted the multiplicity of infection (MOI). The MOI can reach a mean of five clones per infection in high-transmission areas and usually ranges between one and two clones per infected individual in regions of intermediate or low transmission (1). Concurrent clones in multiclone infections are distinguished by genotyping of polymorphic molecular markers. In *in vivo* efficacy drug or vaccine trials in settings where malaria is endemic, parasite genotyping for discrimination of newly incoming versus recrudescent clones after antimalarial treatment is essential for accurate estimation of the efficacy of the intervention (2, 3). Genotyping is further used in malaria molecular epidemiology to assess MOI as a proxy of transmission level in molecular monitoring of interventions and for tracking of individual clones over time in cohort studies to measure the molecular force of infection or duration of infection (4, 5).

The most commonly used P. falciparum molecular markers for genotyping are the length-polymorphic genes encoding the merozoite surface proteins 1 and 2 (*msp1* and *msp2*) and the glutamate-rich protein (*glurp*) (6, 7). The adoption of capillary electrophoresis (CE)-based sizing of PCR products largely resolved limitations of gel-based sizing protocols, such as insufficient resolution of minimal size differences, particularly among fragments larger than 500 bp, or unequal loading of variable PCR yields leading to apparent but artificial size differences. These limitations are particularly relevant for *glurp* genotyping, since amplicons of the *glurp* R2 region often surpass 1,000 bp in size, and CE-based *glurp* genotyping was not routinely used in most laboratories (8, 9).

Parasite clones occasionally remain undetected by PCR despite persisting in a host, an observation described as imperfect clone detectability. The detectability of a clone in an individual sample has been estimated at around 60% in young children from Papua New Guinea (10) and Ghana (5, 11–14) and was found to decrease to 20% in Ghanaian adults older than 60 years (5, 11–14). Imperfect detectability is often attributed to periodical sequestration or fluctuations of parasite densities around the detection limit of PCR as a consequence of naturally acquired immunity to variant antigens. How much technical reasons contribute to imperfect detectability of individual clones has not been investigated systematically for the three prime P. falciparum genotyping markers *msp1*, *msp2*, and *glurp*, but circumstantial observations of clone competition during PCR can be found in the literature (15, 16).

Template competition among differently sized amplicons during PCR as well as the presence of a predominant clone might limit detectability of minority clones in multiclonal infections. This study thus aimed to improve the accuracy of the recommended genotyping procedures for P. falciparum typing routinely applied in malaria drug trials (17) and to establish the dimension of technical limitations in genotyping of recurrent parasitemias. The present findings indicate a dramatic effect of competition during PCR in favor of the smallest fragment.

## RESULTS

### *glurp* genotyping.

**FIG 1:**
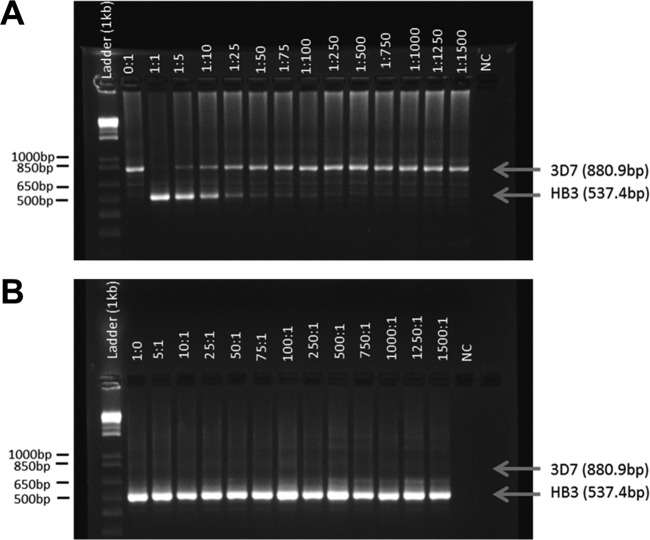
Agarose gel of *glurp* nPCR products obtained from mixtures of P. falciparum *in vitro* culture strains HB3 and 3D7 in different ratios. (A) Strain 3D7 is predominant. (B) Strain HB3 is predominant. NC, negative control. Arrows indicate the sizes of PCR fragments as measured by CE.

We observed significant preferential amplification of the shorter fragment in mixtures of two P. falciparum laboratory strains, 3D7 and HB3, carrying different *glurp* alleles. For the 1:1 ratio, only the shorter HB3 amplicon 537 bp in size was visible after fragment separation on agarose gel (Fig. 1A). The longer 3D7 fragment (881 bp) was only detected if present at least 5-fold in excess over the shorter fragment (Fig. 1A). The HB3 minority clone demonstrated stronger bands than the 3D7 dominant clone up to a HB3/3D7 ratio of 1:10. In the reciprocal dilution series with HB3 as the dominant clone, amplification of the longer 3D7 fragment was completely suppressed by the shorter HB3 amplicon (Fig. 1B). The accuracy and precision of *glurp* sizing were substantially improved by CE compared to agarose gel, but due to the high fluorescence cutoff required to account for characteristic high stutter peaks in *glurp* PCR-CE, the detectability of minority clones was not increased.

### *msp1* and *msp2* genotyping.

**FIG 2:**
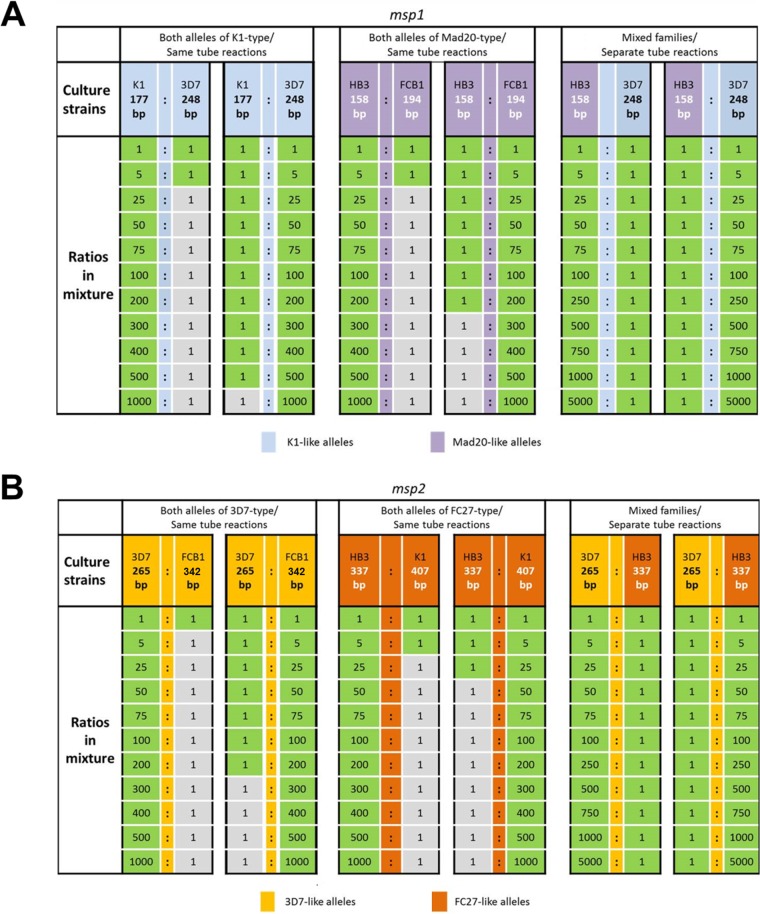
Limit of detection of *msp1* (A) and *msp2* (B) minority clones in mixtures of P. falciparum culture strains. The limit of detection was determined by reciprocal serial dilution of the minority clone. Culture strains carried alleles either of the same allelic family (same tube amplification, left and middle panels) or of different families (amplification in separate tubes, right panels). Green square, allele detected in CE; gray square, allele not detected in CE. Fragments in bp reflect rounded mean allele sizes determined by capillary electrophoresis.

Unlike *glurp*, the alleles of markers *msp1* and *msp2* fall into three and two allelic families, respectively (Table 1). When family-specific *msp1* or *msp2* nested PCRs (nPCRs) were initially multiplexed, pronounced competition between templates was observed irrespective of whether clones belonged to the same or different allelic families. For example, in *msp2* PCR using mixtures of 3D7 (3D7 type) and HB3 (Fc27 type), the minority clone was only detected in ratios up 1:250. To avert such template competition, at least for allele mixtures of different families, all *msp1* and *msp2* nPCRs were performed as simplex family-specific reactions. This strategy increased clone detectability in mixtures of alleles from alternative families drastically, so that the minority clone was detected in all ratios up to 5,000:1 for markers *msp1* and *msp2* (Fig. 2A and B, right panels). However, template competition will still persist if alleles of the same family occur together.

**TABLE 1:** *msp1* and *msp2* allelic families and allele sizes (rounded mean fragment sizes determined by capillary electrophoresis) of the selected P. falciparum *in vitro* culture strains

Strain	Allelic family (allele size [bp])	
	*msp1*^*a*^	*msp2*
HB3	Mad20 type (158)	Fc27 type (337)
3D7	K1 type (248)	3D7 type (265)
K1	K1 type (177)	Fc27 type (407)
FCB1	Mad20 type (194)	3D7 type (342)

a The third *msp1* allelic family, RO33 type, is not length polymorphic and was not included in the analysis of experimental mixtures of culture strains.

**FIG 3:**
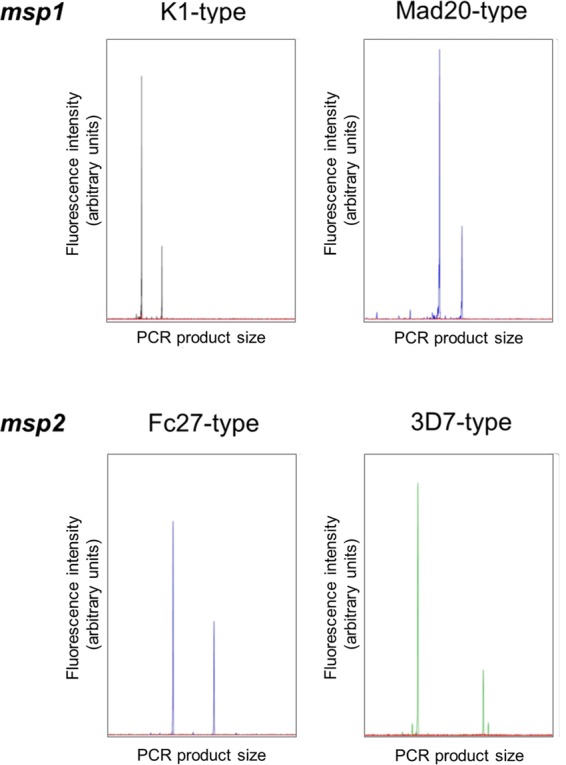
Electropherogram of *msp1* and *msp2* alleles from the same allelic family, amplified from culture strains mixed at a 1:1 ratio.

When two mixed strains carried *msp1* or *msp2* alleles from the same allelic family, the shorter fragment was always preferentially amplified in 1:1 ratios (Fig. 3). The extent of template competition between alleles of the same family was quantified in a systematic analysis of different ratios of two culture strains, which showed that amplicon size had a dramatic effect on PCR outcome (Fig. 2). In mixtures of strains K1 and 3D7, which harbor *msp1* alleles of the same allelic family, the small *msp1* K1 allele (177 bp) was still detected in a 500-fold underrepresentation. In the reciprocal dilution with K1 as the dominant clone, the longer 3D7 allele (248 bp) was only detectable up to a K1/3D7 dilution ratio of 5:1 (Fig. 2A, left panel). A similar picture was observed for the Mad20-type *msp1* family: the shorter HB3 allele was detectable even if 200-fold underrepresented, but vice versa, the longer FCB1 allele was only detectable if it was no more than 5-fold underrepresented (Fig. 2A, middle panel). Results for marker *msp2* mirrored findings from *msp1* in that the shorter allele was preferentially amplified in PCR (Fig. 2B).

CE peak heights did not reflect the ratio of strains mixed (Fig. 3; see Fig. S1 in the supplemental material). The proportions of fluorescent signal from the minority versus dominant clone (Fig. S1), as well as the relative detection limit of a minority clone (Fig. 2), differed between markers and allelic families. *msp2* fragment lengths as well as the size differences between *msp2* alleles were larger than those of *msp1*, and coherently, the detection limit of *msp2* minority clones was reached at lower ratios than *msp1* genotyping (Fig. 2). Not only the size difference between amplicons but also overall PCR fragment size may thus contribute to the observed amplification bias.

### *glurp* clone detectability in mixtures of four culture strains.

**FIG 4:**
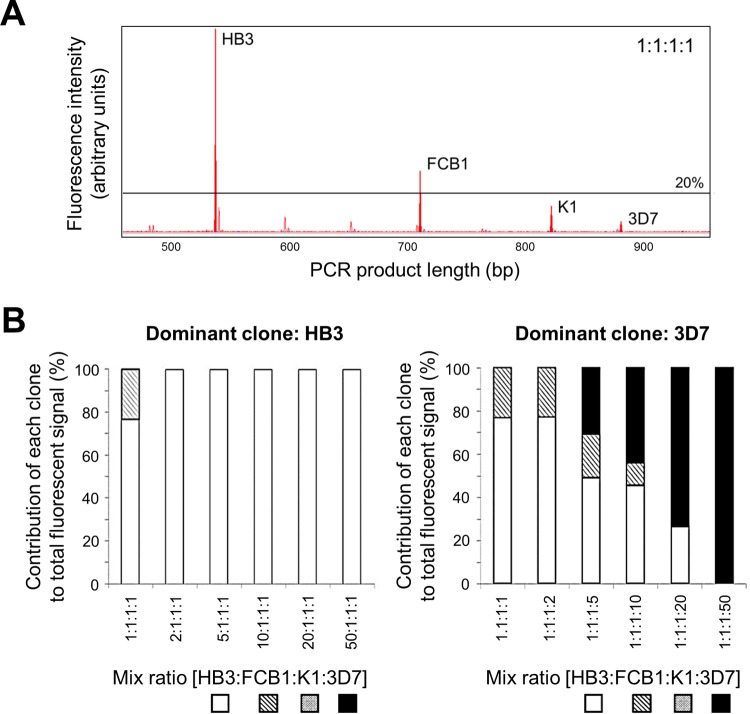
*glurp* minority clone detectability in mixtures of four culture strains. (A) Electropherograms of *glurp* alleles of four P. falciparum culture strains (HB3, FCB1, K1, and 3D7) mixed at a 1:1:1:1 ratio. (B) Proportion of *glurp* fluorescent signal detected during capillary electrophoresis for each clone in a four-culture strain mixture, with clone HB3 (shortest *glurp* allele, left panel) or clone 3D7 (longest *glurp* allele, right panel) as the increasingly dominant clone.

Complex ratios of four culture strains were also assessed. In a 1:1:1:1 mixture of strains, *glurp* fluorescent signal and thus the relative proportion of amplified fragments decreased with increasing amplicon size (Fig. 4A). A 2-fold overrepresentation of the clone with the shortest *glurp* allele (HB3, 537 bp) led to complete suppression of all other fragments after applying the peak height cutoff (Fig. 4B, left panel). In contrast, when strain 3D7 (carrying the longest *glurp* fragment) increased in abundance, it remained undetected until in 5-fold excess over the other clones (Fig. 4B, right panel). The two shortest clones were successively lost only when 25-fold and 50-fold underrepresented toward 3D7 (Fig. 4B, right panel). Intermediate results were obtained when K1 and FCB1 were dominant within the 4-culture strain ratios (see Fig. S2 in the supplemental material).

The extent of template competition between several concurrent clones in *msp1* and *msp2* PCR depends largely on the composition of allelic families in the mixture. For a reliable assessment of amplification bias among more than two alleles per family of *msp1* and *msp2*, a large number of different culture strains would be required.

### Genotyping of field samples.

**FIG 5:**
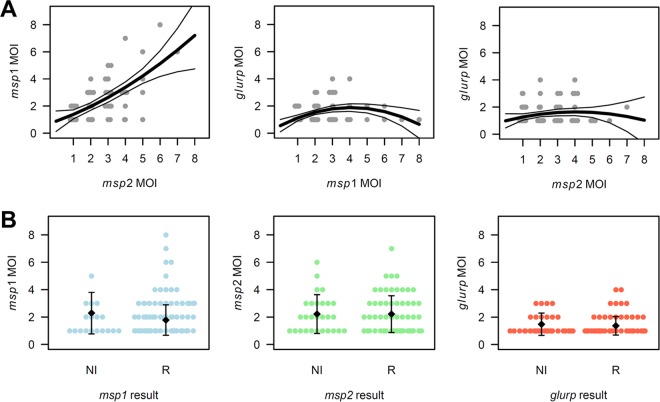
Patterns in *msp1*, *msp2*, and *glurp* MOI in 44 paired pre- and posttreatment samples. (A) Correlation of *msp2*/*msp1*, *glurp*/*msp1*, and *glurp*/*msp2* MOI per sample. Correlations were fitted using 2nd-degree polynomials (thick line) and are shown with 95% confidence intervals (thin lines). (B) *msp1*, *msp2*, and *glurp* MOI per sample classified by genotyping outcome. NI, new infection; R, recrudescence. Mean MOI per class and standard deviation are shown.

Marker performance was compared in genotyping of recurrent parasitemias using 44 paired pre- and posttreatment field samples. The mean *msp2* MOI was slightly higher after simplex allelic family-specific nPCR compared to duplex nPCR (2.2 versus 2.0 clones/infection). With a mean MOI of 1.4 clones per infection, *glurp* consistently identified fewer coinfecting clones compared to markers *msp1* and *msp2* (simplex nPCRs; mean *msp1* and *msp2* MOI of 2.2 clones/infection). Particularly in samples with a high *msp1* or *msp2* MOI, minority clone detection by marker *glurp* was severely limited (Fig. 5A). The mean number of clones detected did not differ between recrudescences and new infections for any marker (Fig. 5B; Wilcoxon *P* value of >0.05).

Genotyping outcomes of all three markers, *msp1*, *msp2*, and *glurp*, agreed for 30/44 (68%) of sample pairs, whereby 22 clear recrudescences and 8 clear new infections were identified (Table 2). When applying the current WHO-recommended approach of typing markers sequentially, all remaining 14 infections were characterized as new infections (Table 2). However, for 7/14 sample pairs, this classification was only supported by questionable marker *glurp*, while both *msp1* and *msp2* denoted a recrudescence. This suggests false classification due to imperfect *glurp* minority clone detectability using the current classification approach. A more appropriate classification would be based on the two better-performing markers *msp1* and *msp2*, which would diagnose these 7 pairs as recrudescence. For the remaining 7/14 sample pairs, markers *msp1* and *msp2* gave disparate results. Two approaches were tested for final classification of these seven sample pairs: first, our proposed approach 1 is based on support from two out of three markers *msp1*, *msp2*, and *glurp*; second, our proposed approach 2 is based on markers *msp1* and *msp2* only, but defining as a decisive characteristic for a new infection a complete switch in allelic family between the pre- and posttreatment samples. In other words, a sample pair was only characterized as a new infection if all alleles detected in the posttreatment sample were different from those in the pretreatment sample and belonged to an allelic family not present in the pretreatment sample. These approaches classified 3/7 (approach 1) or 4/7 (approach 2) sample pairs as recrudescence (see Table S3 in the supplemental material).

**TABLE 2:** Genotyping outcomes for recurrent parasitemias in 44 paired field samples by individual marker and classification into new infection and recrudescence based on combined results

Outcome	*n*^*a*^	Individual marker result^*b*^			Classification^*b*^		
		*msp2*	*msp1*	*glurp*	Current approach^*c*^	Proposed approach 1^*d*^	Proposed approach 2^*e*^
Clear result							
Recrudescence	22	R	R	R	R	R	R
New infection	8	NI	NI	NI	NI	NI	NI
Intermediate result							
Agreement of *msp1* and *msp2*	7	R	R	NI	NI	R	R
Disparate *msp1* and *msp2* result^*e*^	4	NI	R	NI	NI	NI	1 NI, 3 R
	2	NI	R	R	NI	R	1 NI, 1 R
	1	R	NI	R	NI	R	NI
Total no. of:							
Recrudescences					22	32	33
New infections					22	12	11

a *n*, number of sample pairs per pattern.

b NI, new infection; R, recrudescence.

c The current approach to define the overall genotyping result as recommended by WHO uses sequential typing—i.e., stopping the genotyping procedure at first indication of a new infection using markers in the order *msp2*, *msp1*, and *glurp*.

d New approach 1 proposed in this study is based on forming a consensus result: i.e., the result obtained by 2 out of the 3 markers, *msp1*, *msp2*, and *glurp*.

e New approach 2 proposed in this study is based solely on markers *msp1* and *msp2* and compares the compositions of allelic families between pre- and posttreatment samples. A sample pair is only classified as NI if all allelic families differ between between the pre- and posttreatment samples for one of the markers, *msp1* or *msp2*. Detailed genotyping data and classification of these samples are shown in Table S3 in the supplemental material.

In total, 75% (32/44 using approach 1 and 33/44 using approach 2) of recurrent parasitemias after treatment were defined as recrudescent when using the proposed approaches for classification (Table 2). In contrast, only 50% (22/44) would be defined as recrudescent when one marker alone (in 7/14 cases, this was marker *glurp*) was permitted to define a new infection. This suggests misclassification of a third of all recrudescences as new infections by the current approach, mainly due to the marker *glurp*.

## DISCUSSION

The present study allowed investigation of the impact of PCR amplification bias in a controlled environment, revealing a bias more severe than anticipated. A great advantage of shorter over longer fragments was confirmed, with the strongest effect observed for the marker *glurp*. This is likely due to the overall larger *glurp* PCR fragments (within this study, 537 to 1,111 bp) compared to *msp1* (149 to 275 bp) or *msp2* (205 to 506 bp). g*lurp* PCR was also more prone to produce stutter peaks, which cannot easily be distinguished from peaks of minority clones, particularly if a dominant clone is present in a sample. Due to the pronounced stutter peaks in *glurp* PCR, the CE peak height cutoff (applied to prevent mistaking stutter peaks for true fragments) had to be increased for *glurp* PCR compared to *msp1* or *msp2* PCR. As result, true peaks of minority clones are more likely to fall below the cutoff and thus remain undetected in *glurp* PCR, leading to underestimation of MOI.

An essential observation was the importance of performing simplex allelic family-specific nPCRs for both *msp* markers, whenever increased precision is required. The use of allelic family-specific primers to increase sensitivity and resolution of genotyping in samples containing clones from different allelic families has been proposed previously (2, 7, 18). Our results, however, emphasize that unless nPCRs are performed separately, the gain in minority clone detectability using allelic family-specific primers is marginal. A great gain in sensitivity is observed when nPCRs are performed separately, which justifies the increase in diagnostic cost. Yet, template competition between alleles from the same family cannot be overcome even by simplex nPCR. Marker *glurp* with only one allelic family and longest amplicon sizes can thus be considered the least suitable marker for sensitive discrimination of clones. Studies performed before the adoption of CE-based genotyping methods used PCR-restriction fragment length polymorphism (PCR-RFLP) to distinguish *msp2* clones (19). This protocol did not use family-specific *msp2* primers; therefore, all amplified fragments (ranging from 350 to 750 bp) likely were also subject to amplification bias. Yet, fragments on average were substantially smaller than *glurp* fragments, which ranged from 537 to 1,111 bp in this study.

The significantly lower MOI observed in our field samples using marker *glurp* compared to *msp1* and *msp2* could be due to a lower diversity of *glurp*, but a major factor seems to be suppression of minority clones or long fragments during *glurp* PCR. In light of the severe bias in *glurp* PCR in artificial mixtures of culture strains, new infections identified only by *glurp* genotyping and not by the other two markers are probably attributable to competition in PCR and should be interpreted with care. It is therefore crucial to consider PCR limitations such as amplification bias when designing a strategy for genotyping and data analysis.

The WHO definition of a recrudescent infection is the presence of at least one shared genotype in the compared pre- and posttreatment samples at all loci. A new infection is defined by the presence of only new alleles in the posttreatment sample by at least one of the markers (3). The effects of intra-allelic family competition during PCR demonstrated here suggest a reconsideration of the current recommended genotyping procedure, which stipulates consecutive analysis of the three markers. According to the current strategy, once a new infection is observed by one of the markers the remaining loci are not typed. In contrast, if amplification of both *msp* markers with family-specific simplex nPCRs was mandatory, minority clone detectability would be drastically increased due to (i) the lower likelihood of two clones to harbor alleles of the same *msp1* as well as the same *msp2* family, and (ii) the independence of allele sizes of *msp1* and *msp2* for any given clone, making it unlikely that alleles of both markers will be suppressed during PCR.

Marker *glurp*, despite its shortfalls, might be useful until a more suitable marker is found and validated, as it can provide additional resolution in areas of low transmission with only limited diversity in both *msp* markers. In contrast, in study areas with high mean MOI, *glurp* likely contributes to overestimation of new infections, as later recrudescing minority clones could easily be missed in a multiclonal admission sample. The use of *glurp* as a third marker should hence be critically reviewed, and its application could be restricted to drug trials performed in low-endemicity settings. Furthermore, a more robust definition of “new infection” should be discussed among experts, which could be primarily, or exclusively, based on markers *msp1* and *msp2*. In practice, genotyping of recurrent parasitemias should therefore begin with typing both markers *msp1* and *msp2*. If these two markers agree, *glurp* genotyping would no longer be necessary. In case of a disagreement between *msp1* and *msp2* genotyping outcomes, either *glurp* typing would have to be performed and the “two out of three” rule applied (preferred in low-endemicity settings), or the composition of allelic *msp1* and *msp2* families would be considered (preferred in high-endemicity settings). In the latter approach, final classification as a new infection would require a complete shift of allelic families between pre- and posttreatment samples for either *msp1* or *msp2*, as clone competition is abrogated between allelic families if nPCRs are performed as simplex PCRs.

In summary, the following alterations of the current genotyping strategy could be considered: (i) separate nPCRs for each allelic family, (ii) obligate genotyping of the two markers *msp1* and *msp2*, or (iii) classification of recurrent parasitemias based on a consensus result of markers *msp1* and *msp2* first, with disparate *msp1* and *msp2* results resolved using marker *glurp* or, as potentially the best option, by rating *msp1*/*msp2* allelic families for defining a new infection.

In areas where malaria is highly endemic, with frequent multiclone infections, these amendments could affect the outcome of drug efficacy trials in that less recrudescent infections would be misclassified as new infections, and in consequence, overestimation of the efficacy of a tested drug would be reduced.

In the future, such technical limitations might be overcome by the development of single-nucleotide polymorphism-based genotyping techniques, which, in combination with molecular barcoding or next-generation sequencing, might provide highly diverse haplotype markers with sufficient resolution for tasks such as genotyping of recurrent parasitemias (20, 21).

The detectability of a clone in a blood sample is determined by several factors, such as sensitivity of the molecular assay, biological features of the parasite (i.e., sequestration of synchronous clones and fluctuation of densities), and technical limitations. The contribution of each of these factors to a final genotyping result is challenging to disentangle. The improvements suggested for recrudescence typing will not lead to perfect detectability of parasite clones. This method will also not question previous results from earlier applications of genotyping in *in vivo* drug efficacy trials, but it provides a rational basis for a genotyping approach that is less prone to false outcomes by correcting a technical problem.

The WHO/Medicines for Malaria Venture (MMV) consultation (3) had suggested that genotyping should be performed in a sequential manner, starting with marker *msp2* or *glurp*, and then as a third marker, *msp1* was suggested. This decision was based on the workload incurred by markers for which more than one nested PCR needs to be performed. According to the present data, using *glurp* as the first marker in sequential genotyping likely leads to a stronger bias toward a “new infection.” However, in many laboratories, *msp2* followed by *msp1* were used as the first and main markers. Therefore, our suggestion of a new genotyping approach will not dramatically change the PCR-corrected outcomes in future clinical trials compared to those of the past, but the precision will be improved. Despite the known shortfalls in genotyping, its application in field trial is indispensable, as in areas of intermediate transmission superinfections during the follow-up period of the trial will yield an incorrect high drug failure rate. In high-transmission settings, the numbers of superinfections are high and clone competition in PCR will be more dramatic. Thus, genotyping is bound to be more accurate than in areas of low and intermediate transmission. However, as multiplicity of infection is declining in most areas of endemicity in the world, the value of genotyping will increase in the future, as samples from the clinical trial areas will have fewer concurrent infections and thus less clone competition.

In conclusion, this study highlights major limitations of P. falciparum genotyping using length-polymorphic markers, which are caused by fragment size differences and intra-allelic family competition during nPCR. Our data suggest inclusion of both *msp* markers for genotyping of recurrent parasitemias and raises questions on the use of additional *glurp* genotyping. Relying solely on *glurp* genotyping should be discouraged due to the absence of allelic families and therefore direct template competition between all alleles. Small adjustments to the currently recommended genotyping procedures for clinical trials could significantly improve the accuracy of PCR correction of endpoints in malaria drug or vaccine efficacy trials.

## MATERIALS AND METHODS

### Culture strains and field samples.

Four Plasmodium falciparum culture strains, HB3, 3D7, K1, and FCB1, were selected to represent the allelic families of *msp1* and *msp2* (Table 1). DNA extractions from cultured parasites and blood from a human noninfected volunteer were performed using the QIAamp DNA blood minikit (Qiagen, Germany), and the DNA was stored at 4°C. Parasites were quantified by quantitative PCR targeting the P. falciparum 18S rRNA gene using a serial dilution of ring-stage parasites as quantification standards (22, 23). DNA from 2 or 4 strains was mixed in human DNA, to reconstitute human blood, in ratios ranging from 1:1 to 5,000:1. For the minority clone, a minimal concentration of 10 parasite genomes per microliter of blood was chosen to prevent stochastic effects in positivity due to insufficient template in the PCR.

Genotyping was performed on 44 paired anonymous field samples. A sample pair consisted of a pretreatment sample and a sample collected on the day of recurrent parasitemia after antimalarial treatment. Mean time to recurrence was 14 days on average (range, 6 to 28 days). These samples were derived from clinical drug efficacy trials and had been collected at several sites across Africa and Asia. Therefore, a description of “overall” endemicity is not possible for the set of samples used. As a surrogate measure of transmission intensity, the mean multiplicity of infection (MOI) in baseline samples represents a useful parameter in the context of the present article. The mean MOI for marker *msp2* at baseline was 2.3 concurrent infections per PCR-positive individual, with individual MOI ranging from 1 to 7 infections. Ethical clearance for genotyping of these anonymized blood samples was obtained from the Ethikkomission Nordwest-und Zentralschweiz (EKNZ Req-2016-00050).

### Genotyping PCR and CE.

Primer sequences, PCR mixtures, and cycling conditions are listed in Tables S1 and S2 in the supplemental material. The *msp1* and *msp2* primers correspond to the WHO recommended primers (17), while *glurp* primary PCR (pPCR) and nPCR primers were optimized for CE in the present study. Allelic family-specific simplex and multiplex nPCRs were compared for marker *msp2* using DNA from culture strain mixtures and field samples. Unless stated otherwise, results from allelic family-specific simplex nPCRs are reported. CE of markers *msp1* and *msp2* used the GeneScan 500 LIZ dye size standard (Thermo Fisher Scientific) on an AB3130xl automated sequencer. *glurp* fragment sizing was performed at Macrogen Corp (Amsterdam, The Netherlands) using the GeneScan 1200 LIZ dye size standard (Thermo Fisher Scientific). Fragment analysis was performed with GeneMapper software version 5 (Applied Biosystems). A peak height cutoff for minority clones was set at 10% of the height of the dominant peak for *msp1* and *msp2* genotyping. For genotyping of *glurp*, the cutoff was increased to 20% of the dominant peak to account for the characteristic high stutter peaks.

## Supplementary Material

Supplemental material
