# Supplementary material for: Critical Evaluation of Molecular Monitoring in Malaria Drug Efficacy Trials and Pitfalls of Length-Polymorphic Markers
Source: Antimicrob Agents Chemother. 2016 Dec 27;61(1):e01500-16. doi: 10.1128/AAC.01500-16 (PMC5192142; doi:10.1128/AAC.01500-16)
Supplement: Supplemental material [file supp_61_1_e01500-16__index.html]

Critical Evaluation of Molecular Monitoring in Malaria Drug Efficacy Trials and Pitfalls of Length-Polymorphic Markers — Supplemental material 

# Critical Evaluation of Molecular Monitoring in Malaria Drug Efficacy Trials and Pitfalls of Length-Polymorphic Markers

## Supplemental material

- Supplemental file 1 -

  Figure S1 and S2 and Tables S1, S2, and S3

  PDF, 901K
