# Supplementary material for: Critical Evaluation of Molecular Monitoring in Malaria Drug Efficacy Trials and Pitfalls of Length-Polymorphic Markers
Source: Antimicrob Agents Chemother. 2016 Dec 27;61(1):e01500-16. doi: 10.1128/AAC.01500-16 (PMC5192142; doi:10.1128/AAC.01500-16)
Supplement: Supplemental material [file AAC.01500-16_zac001175834s1.pdf]

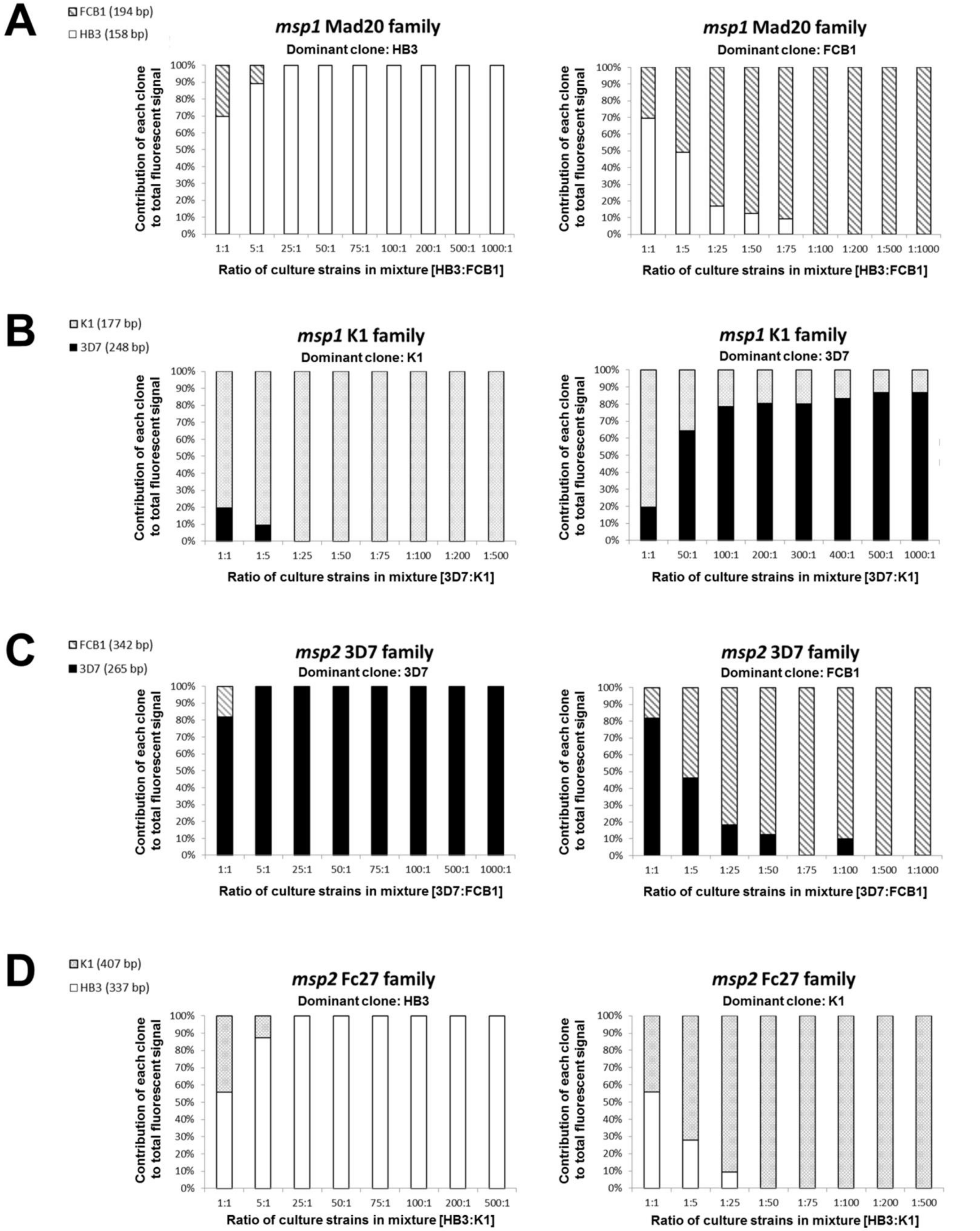

Supplementary Figure S1: **Proportion of *msp1* and *msp2* fluorescent signals detected during capillary electrophoresis for each clone in two culture strain-mixtures.** Mixed strains carried *msp1* or *msp2* alleles of the same allelic family: (A) Mad20-type *msp1* alleles, (B) K1-type *msp1* alleles, (C) 3D7-type *msp2* alleles, and (D) FC27-type *msp2* alleles. The shorter allele was increasingly dominant in the left panels, the longer allele increasingly dominant in the right panels.

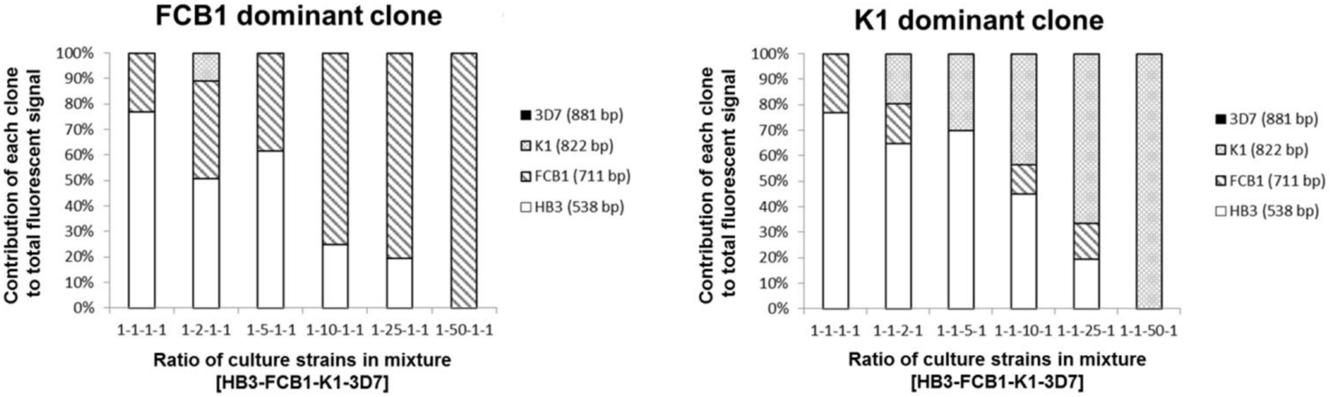

Supplementary Figure S2: Proportions of *glurp* fluorescent signal detected during capillary electrophoresis for each clone in a four culture strain-mixture, with clone FCB1 (2nd-shortest *glurp* allele, left panel) or clone 3D7 (2nd-longest *glurp* allele, right panel) as increasingly dominant clone.

Supplementary Table S1: PCR reaction mixes and thermocyclic profiles

A) PCR genotyping *glurp*

Primary PCR reaction mix and thermo profile

Reagents	Concentration	Volume
ddH2O		25.5 µL
10x Buffer B	10x	5.0 µL
dNTPs	2mM	5.0 µL
$MgCl_2$	25mM	4.0 µL
Primer G4	10µM	2.5 µL
Primer G5mod	10µM	2.5 µL
FIRE Pol®		0.5 µL
DNA		5 µL
		50 µL

Temperature	Time	Cycles
94°C	5min	1x
94°C	1min	
50°C	1min	25x
72°C	2min	
72°C	10min	1x

Nested PCR reaction mix and thermo profile

Reagents	Concentration	Volume
ddH2O		31.5 µL
10x Buffer B	10x	5.0 µL
dNTPs	2mM	5.0 µL
$MgCl_2$	25mM	3.0 µL
GNF	10µM	2.0 µL
G3	10µM	2.0 µL
FIRE Pol®		0.5 µL
pPCR product		1 µL
		50 µL

Temperature	Time	Cycles
94°C	5min	1x
94°C	1min	
58°C	1min	30x
72°C	1min	
72°C	10min	1x

FIRE Pol® (Solis BioDyne, England)

B) PCR genotyping *msp1/2*

Primary PCR reaction mix and thermo profile (duplex msp1 & msp2)

Reagents	Concentration	Volume
ddH2O		28.0 µL
10x Buffer B	10x	5.0 µL
dNTPs	2mM	5.0 µL
$MgCl_2$	25mM	4.0 µL
Primer Mix M1/M2[a]	10µM	2.5 µL
FIRE Pol®		0.5 µL
DNA		5 µL
		50 µL

Temperature	Time	Cycles
94°C	5min	1x
94°C	30sec	
54°C	1min	30x
72°C	1min	
72°C	5min	1x

[a] Primer mix: M1-OF/M1-OR/M2-OF/M2-OR

msp1 nested PCR reaction mix and thermo profile

Reagents	Concentration	Volume
ddH2O		32.5 µL
10x Buffer B	10x	5.0 µL
dNTPs	2mM	5.0 µL
$MgCl_2$	25mM	4.0 µL
Primer Mix nPCR[b]	10µM	2.0 µL
FIRE Pol®		0.5 µL
pPCR product		1 µL
		50 µL

Temperature	Time	Cycles
94°C	5min	1x
94°C	30sec	
59°C	1min	30x
72°C	1min	
72°C	5min	1x

[b] Primer mix: M1-MF/M1-MR or M1-KF/M1-KR

msp2 nested PCR reaction mix and thermo profile

Reagents	Concentration	Volume
ddH2O		33.5 µL
10x Buffer B	10x	5.0 µL
dNTPs	2mM	5.0 µL
$MgCl_2$	25mM	3.0 µL
Primer Mix nPCR[c]	10µM	2.0 µL
FIRE Pol®		0.5 µL
pPCR product		1 µL
		50 µL

Temperature	Time	Cycles
94°C	5min	1x
94°C	30sec	
50°C	45sec	30x
72°C	1min	
72°C	10min	1x

[c] Primer mix: M5/S-tail or N5/S-tail

Supplementary Table S2: PCR primers

A) glurp genotyping PCR

	Primer ID	T_m	Sequence	CG%	nt	Reference
primary PCR	G4	57.4°C	5'-acatgcaagtgttgatcc-3'	44.4	18	
	G5mod	64.2°C	5'-cagatggtttgggagtaacgtt-3'	45.5	22	
nested PCR	G3	64°C	5'-tgtaggtaccacgggttcttg-3'	52.4	21	
	GNF	65.5°C	(PET) 5'-tgttcacactgaacaattagatttagatca-3'	30	30	Snounou (2002)

B) msp1 genotyping PCR

	Primer ID	T_m	Sequence	CG%	nt	Reference
primary PCR	M1-OF	61.9°C	5'-ctagaagctttagaagatgcagtattg-3'	37	27	Snounou (2002)
	M1-OR	61.1°C	5'-cttaaatagtattctaattcaagtggatca-3'	26.7	30	Snounou (2002)
nested PCR						
Mad20 family	M1-MF	70.1°C	Tail 5'-aaatgaaggaacaagtggaacagctgttac-3'	40	30	Snounou (2002)
	M1-MR	67.6°C	(FAM) 5'-atctgaaggatttgtacgtcttgaattacc-3'	36.7	30	Snounou (2002)
K1 family	M1-KF	65.3°C	Tail 5'-aaatgaagaagaaattactacaaaaggtgc-3'	30	30	Snounou (2002)
	M1-KR	83.2°C	(NED) 5'-gcttgcatcagctggagggcttgcaccaga-3'	60	30	Snounou (2002)

C) msp2 genotyping PCR

	Primer ID	T_m	Sequence	CG%	nt	Reference
primary PCR	M2-OF	58.9°C	5'-atgaaggtaattaaaacattgtctattata-3'	20	30	Snounou (2002)
	M2-OR	62.2°C	5'-ctttgttaccatcggtacattctt-3'	37.5	24	Snounou (2002)
nested PCR						
FC27 family	S-tail-fw	54.3°C	Tail 5'-gcttataatatgagtataaggagaa-3'	28	25	Falk et al. (2006)
	M5-rev	52.2°C	(FAM) 5'-gcattgccagaacttgaa-3'	47.4	19	Falk et al. (2006)
3D7 family	S-tail-fw	54.3°C	Tail 5'-gcttataatatgagtataaggagaa-3'	28	25	Falk et al. (2006)
	N5-rev	60.5°C	(VIC) 5'-ctgaagaggtactggtaga-3'	44.4	18	Falk et al. (2006)

Supplementary Table S3: Genotyping data and classification of 7 samples with discrepant *msp1/msp2* result

Sample pair	*msp1*			*msp2*			*glurp*			Classification	
	D0	DX	Result	D0	DX	Result	D0	DX	Result	Proposed Approach 1	Proposed Approach 2
1	M211	K238<br>RO33	NI<br>with shift of allelic families	D226<br>D370	D370<br>F417	R	766<br>933	766	R	**R**	**NI**
2	K230<br>K238<br>Ro33	Ro33<br>K212	R	D257<br>D298<br>F371	D269	NI	939	819<br>884	NI	**NI**	**R**
3	Ro33	Ro33	R	F417	D224	NI<br>with shift of allelic families	773	884	NI	**NI**	**NI**
4	M219<br>M202<br>K177<br>K203<br>K212<br>K230<br>K284<br>Ro33	M219<br>K212<br>Ro33	R	D226<br>D234<br>D271<br>D282<br>D291<br>F408	D241<br>D250<br>D375<br>F337<br>F382	NI	602	832	NI	**NI**	**R**
5	K203<br>K212<br>Ro33	Ro33<br>K257	R	D286<br>D352	F337	NI<br>with shift of allelic families	939	939<br>1111	R	**R**	**NI**
6	M193<br>M202<br>K239<br>Ro33	Ro33<br>K194<br>K212	R	D253<br>F371<br>F454	D343<br>F348<br>F419	NI	822<br>939	831<br>1052<br>1111	NI	**NI**	**R**
7	M193	M193	R	D300<br>F371	F337	NI	602<br>830	602	R	**R**	**R**
